# Genotypic characterisation and cluster analysis of *Campylobacter jejuni *isolates from domestic pets, human clinical cases and retail food

**DOI:** 10.1186/2046-0481-64-6

**Published:** 2011-03-31

**Authors:** Els Acke, Cyril Carroll, Aoife O'Leary, Kevina McGill, Lorraine Kelly, Amanda Lawlor, Robert H Madden, Lynn Moran, Pam Scates, Eleanor McNamara, John E Moore, Boyd R Jones, Seamus Fanning, Paul Whyte

**Affiliations:** 1 Centre for Food Safety, School of Agriculture, Food Science and Veterinary Medicine, University College Dublin, Belfield, Dublin 4, Ireland; 2 Small Animal Clinical Studies, School of Agriculture, Food Science and Veterinary Medicine, University College Dublin, Belfield, Dublin 4, Ireland; 3 Enteric Pathogen Research Laboratory, Department of Microbiology, National University of Ireland, Galway, Ireland; 4 Agri-Food and Biosciences Institute, Newforge Lane, Belfast, Northern Ireland, UK; 5 Public Health Laboratory, Cherry Orchard Hospital, Ballyfermot, Dublin 10, Ireland; 6 Northern Ireland Public Health Laboratory, Department of Bacteriology, Belfast City Hospital, Belfast BT9 7AD, Northern Ireland, UK; 7 Veterinary Teaching Hospital, Institute of Veterinary, Animal and Biomedical Sciences, Private Bag 11 222 Massey University, Palmerston North 4442, New Zealand

## Abstract

The genetic similarity of *Campylobacter jejuni *isolates from pets, compared to human clinical cases and retail food isolates collected in Ireland over 2001-2006 was investigated by cluster analysis of pulsed-field gel electrophoresis (PFGE) fingerprinting profiles. Comparison of the PFGE profiles of 60 pet isolates and 109 human isolates revealed that seven (4.1%) profiles were grouped in clusters including at least one human and one pet *C. jejuni *isolate. In total six (1.6%) of 60 pet and 310 food profiles were in clusters with at least one food and one pet *C. jejuni *isolate. The detection of only a small number of genetically indistinguishable isolates by PFGE profile cluster analysis from pets and from humans with enteritis in this study suggests that pets are unlikely to be an important reservoir for human campylobacteriosis in Ireland. However, genetically indistinguishable isolates were detected and *C. jejuni *from pets may circulate and may contribute to clinical infections in humans. In addition, contaminated food fed to pets may be a potential source of *Campylobacter *infection in pets, which may subsequently pose a risk to humans.

## Background

A major source of *Campylobacter *infections in humans is the handling or consumption of contaminated meat, especially poultry. Other risk factors for infection include ingestion of contaminated dairy products (for example unpasteurised milk), drinking contaminated water, contact with pets, foreign travel, and swimming in natural sources of water [1-3]. It is estimated that *C. jejuni *accounts for approximately 80-95% of all enteric *Campylobacter *infections in humans, with *C. coli*, *C. lari*, *C. upsaliensis *and *C. fetus *less commonly isolated [4,5]. Many questions on the epidemiology of *Campylobacter *spp. remain unanswered due to the high degree of genetic diversity observed in *Campylobacter *spp. populations [6,7], the low number of isolates in which speciation and subtyping is performed [8], the under reporting of cases because of the self-limiting nature of *Campylobacter *infections in most individuals [9], and the fact that most cases of human campylobacteriosis are sporadic as opposed to large outbreaks [10]. Contact with pets has been recognised as a risk factor for campylobacteriosis in humans [1,11,12]. Pets have been shown to be carriers of *Campylobacter *spp. with *C. upsaliensis*, *C. jejuni *and *C. helveticus *the predominant species isolated [13-16]. Although *C. upsaliensis *was the most commonly isolated *Campylobacter *spp. from household pets in Ireland with a prevalence of 65%, *C. jejuni *was also frequently isolated (22.5%) [16]. Wolfs *et al. *[17] reported the first genetically proven case of *C. jejuni *transmission between pets and humans in a 3-week-old infant who acquired the infection from a recently obtained household puppy with diarrhoea. Damborg *et al. *[18] reported the occurrence of identical *C. jejuni *strains in a 2-year-old girl and her dog in Denmark confirmed by PFGE. With reported *Campylobacter *spp. isolation rates of up to 87% and 75% in dogs and cats respectively in Ireland [15,16], pets could play a substantial role in the epidemiology of campylobacteriosis. The aim of this retrospective study was to compare the genotypes from pet *C. jejuni *isolates to a large number of human clinical and retail food *C. jejuni *genotypes obtained on the island of Ireland and to assess if isolates from different sources were indistinguishable, based on PFGE profile cluster analysis.

## Methods

*C. jejuni *isolates from pets included in this study (n = 60) were collected from Irish cats and dogs in shelters and private households as part of a large *Campylobacter *spp. prevalence study. The majority of cats and dogs sampled lived in the Dublin area and Belfast. Healthy pets and pets with signs of gastro-intestinal disease were included in the study [7,15,16]. One rectal swab was obtained from each animal and swabs were cultured using a range of specialised culture methods to optimise recovery of *Campylobacter *spp. [19]. DNA was extracted and *Campylobacter *speciation was performed by PCR analysis at the Centre for Food Safety at University College of Dublin (UCD) as previously described [15]. As part of a Food Safety Promotion Board-funded (FSPB) project, retail food samples were collected from a range of supermarkets and butcher shops in three regions in Ireland: Dublin, Galway and Belfast. The majority of the samples included in the present study were from poultry meat. Sampling procedures and *Campylobacter *isolation techniques used for food analysis are described by Whyte *et al. *[20]. Concurrently with the isolation of the food isolates, *Campylobacter *isolates from human clinical cases were submitted by collaborating Public Health Laboratories in Dublin, Galway and Belfast. These isolates were obtained from patients who had presented to either general practitioners or had been admitted to hospitals with signs of gastro-enteritis. All *C. jejuni *human clinical, food and pet isolates were collected between 2001-2006. Genotyping was performed using PFGE at the Enteric Pathogen Research Laboratory, National University of Ireland, Galway. All *C. jejuni *isolates obtained from pets were genotyped using identical methods at the Centre for Food Safety, UCD. PFGE profiling of the *C. jejuni *isolates was carried out using the standardised 'Campynet' protocol as previously described [21]. The restriction enzyme used was *Sma*I (R6125, ProMega, USA). PFGE gels obtained after electrophoresis were captured in a TIFF format by using a GelDoc 1000 high-resolution image capture system (Bio-Rad).

A database of genotypes of the pet, retail food and human clinical *C. jejuni *isolates was subsequently assembled at the Centre for Food Safety, UCD [22,23]. PFGE profiles were analysed using Fingerprinting II software, Bionumerics Version 3.0 (Applied Maths, Kortrijk, Belgium). A combination of Dice and UPGMA (unweighted paired group method with arithmetic mean), position tolerance value of 1.5% and an optimisation value of 1.0% was used [24]. Clusters were defined as isolates with at least 90% genetic similarity. These isolates were considered indistinguishable.

## Results and Discussion

The genotypic relatedness of *C. jejuni *isolates obtained from pets and those obtained from human clinical and retail food samples in Ireland were determined by PFGE profile cluster analysis. Cluster analysis of 60 pet *C. jejuni *profiles compared to 109 human clinical *C. jejuni *profiles showed 4.1% of isolates were in clusters containing at least one pet and one human isolate. One cluster of three included two isolates from adults with gastroenteritis in Belfast and an adult dog with no signs of gastro-intestinal disease from the Dublin area. There were two clusters each containing two isolates; one of these contained isolates from an adult with gastro-enteritis in Galway and from a healthy shelter puppy in the Dublin area. The other cluster contained isolates from an adult with gastro-enteritis in Galway and a household puppy from Dublin with diarrhoea. The occurrence of genetically indistinguishable *C. jejuni *isolates appears to be uncommon when Irish pet isolate profiles were compared to human isolate profiles. The present study included isolates from different sources collected over several years and in different regions of Ireland, making it less likely that common isolates were present and further investigations on pets as a potential reservoir for human campylobacteriosis are indicated. The true prevalence world wide of indistinguishable isolates occurring in pets and humans may be underestimated due to a number of factors including the under reporting of infections, lack of epidemiological studies including animals, the occurrence of mixed *C. jejuni *populations, intermittent excretion of campylobacters [25], and the high level of genetic diversity in *C. jejuni *populations [7,26]. The study design plays an important role and no large-scale studies have been performed to date evaluating human clinical cases of campylobacteriosis concurrently with *Campylobacter *spp. detected from pets in their environment. Additional epidemiological links could also exist between *C. jejuni *isolates from different sources if other enzymes for PFGE are used, or due to small genetic rearrangements in the organisms [18,27].

**Figure 1:**
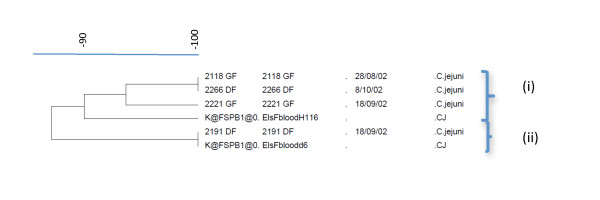
**Clusters of *C. jejuni *isolates**. (i) A cluster of four with a dog isolate (H116), and three food sample isolates (2118GF, 2266DF, 2221GF) and (ii) a cluster of two with a food sample isolate (2191DF) and a shelter puppy isolate (d6). The scale indicates percentage genetic similarity.

The handling and consumption of contaminated meat, especially poultry, has long been implicated as a major source of campylobacteriosis in humans [3,10,28]. Food may also play an important role in *Campylobacter *spp. colonisation in pets, which may consequently result in human infection [29], and food as a common source of infection in pets and humans could be possible. Also drinking unpasteurised milk or contaminated water, and direct (fresh faeces) or indirect (contaminated food bowls, environment) contact with infected animals can be a source of infection in pets [29]. Genotypic comparisons of *C. jejuni *isolate PFGE profiles from pets to a large number of food isolate profiles has not been reported previously. Cluster analysis of 60 pet *C. jejuni *profiles compared to 310 retail food *C. jejuni *profiles obtained by PFGE showed 1.6% of isolates were in clusters. One cluster of four containing an isolate from cow's liver sampled in Galway, an isolate from lamb's heart sampled in Dublin, a Galway chicken isolate and an isolate from an adult dog with no signs of gastro-intestinal disease from the Dublin area. One cluster of two isolates was detected comprising a Dublin chicken isolate and an isolate from a healthy shelter puppy in the Dublin area (Figure 1). The two pet isolates that clustered with the food isolates were the same as two out of three that clustered with the human clinical isolates. With the low number of pet and food isolates that clustered based on PFGE profile analysis, retail food appears an unlikely source of *Campylobacter *infections in pets. However, the feeding habits in the households and shelters where the pets lived were unknown and a possible direct link between feeding contaminated retail foods to pets and *Campylobacter *spp. isolation was not assessed in the present study.

The epidemiology of campylobacteriosis is highly complex due to the many animal and environmental reservoirs [30]. In addition, a high level of genetic diversity between isolates from pets has now also been confirmed [7,26]. Since *Campylobacter *spp. are zoonotic pathogens and asymptomatic animals could act as reservoirs, pets in contact with people with increased risk of infection (e.g., immunocompromised children) may represent an important health risk and adequate hygiene practices in households with pets are essential [31]. Adequate hygiene measures are required also when feeding pets meat or other potentially contaminated foods. Further studies involving larger populations, a variety of sampling groups, testing for multiple *Campylobacter *spp., and specialised molecular techniques are indicated to clarify the role of pets in human campylobacteriosis and to improve the understanding of the complex epidemiology of *Campylobacter *infections.

## Conclusions

The detection of indistinguishable PFGE profiles of *C. jejuni *obtained from pets, human clinical cases and retail food samples in different geographical regions on the island of Ireland, collected over several years, suggests that isolates may circulate between different sources. The presence of a small number of clusters containing isolates from dogs and human clinical cases makes it unlikely that pets represent a substantial risk for human campylobacteriosis, however, further investigations aimed specifically at confirmation of zoonotic transmission are required.

## Competing interests

The authors declare that they have no competing interests.

## Authors' contributions

EA drafted the work, performed pet sample collection, bacterial cultures and molecular work including PFGE of *C. jejuni *and the cluster analysis of the pet isolates, CC and AOL performed the sample collection, bacterial cultures and molecular work including PFGE of the Galway human clinical and retail food isolates, KMG and EMN participated in bacterial cultures and molecular work of Dublin pet, human clinical and retail food isolates, LK performed the cluster analysis of the *C. jejuni *isolates obtained from human clinical cases and retail food and assembled the database at the Centre for Food safety, AL participated in the PFGE of pet isolates, RHM, LM, PS, and JEM performed the sample collection, bacterial cultures and molecular work including PFGE of the Belfast human clinical and retail food isolates BRJ, SF and PW were involved in the revision of the manuscript, the project design and the analysis and interpretation of the data assembled in this work. All authors read and approved the final manuscript.
